# The Associations between Serum Zinc Levels and Metabolic Syndrome in the Korean Population: Findings from the 2010 Korean National Health and Nutrition Examination Survey

**DOI:** 10.1371/journal.pone.0105990

**Published:** 2014-08-25

**Authors:** Jin-A Seo, Sang-Wook Song, Kyungdo Han, Kyung-Jin Lee, Ha-Na Kim

**Affiliations:** 1 Department of Family medicine, St. Vincent’s Hospital, College of Medicine, The Catholic University of Korea, Seoul, Korea; 2 Department of Biostatistics, College of Medicine, The Catholic University of Korea, Seoul, Korea; Department of Preventive Medicine, College of Medicine, The Catholic University of Korea, Seoul, Korea; Innsbruck Medical University, Austria

## Abstract

The prevalence of metabolic syndrome has been increasing rapidly worldwide. The functions of zinc may have a potential association with metabolic syndrome, but such associations have not been investigated extensively. Therefore, we examined the relationship between serum zinc levels and metabolic syndrome or metabolic risk factors among South Korean adults ≥20 years of age. The analysis used data from the Korean National Health and Nutrition Examination Survey, a cross-sectional survey of Korean civilians, conducted from January to December 2010. A total of 1,926 participants were analyzed in this study. Serum zinc levels in men were negatively associated with elevated fasting glucose (adjusted odds ratio [aOR], 0.58; 95% confidence interval [CI], 0.36–0.93) and positively associated with elevated triglycerides (aOR, 1.47; 95% CI, 1.01–2.13). A difference in serum zinc levels was detected in women, depending on the number of metabolic syndrome components (p = 0.002). Furthermore, serum zinc levels showed a decreasing trend with increasing numbers of metabolic syndrome components in women with metabolic syndrome. These findings suggest that serum zinc levels might be associated with metabolic syndrome or metabolic risk factors. Further gender-specific studies are needed to evaluate the effect of dietary or supplemental zinc intake on metabolic syndrome.

## Introduction

Zinc is the second most common trace metal in the body and, as an essential micronutrient, is important in growth and development. Zinc also has crucial roles in the synthesis, storage and secretion of insulin and in the actions of insulin on carbohydrate metabolism [1], [2]; thus, zinc possesses an insulinomimetic effect [3], [4]. Furthermore, zinc plays vital roles as a cofactor for metalloenzymes in antioxidant defense systems such as those involving superoxide dismutase, catalase, and glutathione peroxidase [5], and as reducing inflammatory cytokine production via regulation of a zinc-finger protein [6], [7].

Metabolic syndrome (MetS), a cluster of metabolic risk factors including hyperglycemia, atherogenic dyslipidemia, elevated blood pressure and abdominal obesity, is associated with an increased risk of cardiovascular disease and all-cause mortality [8]–[10]. The prevalence of MetS has been increasing rapidly worldwide [11] such that MetS has become a major medical issue. The prevalence of MetS in U.S. adults was 27.9% according to the National Health and Nutrition Examination Survey (NHANES) of 1988–1994 and 34.1% according to the NHANES of 1999–2006 [12]. In Korea, the prevalence of MetS increased from 24.9% in 1998 to 31.3% in 2007, according to the Korean National Health and Nutrition Examination Survey (KNHANES) [13].

MetS represents a complex interaction of maladaptive characteristics related to impaired insulin action at target organs, suggesting that insulin resistance plays a key role in the pathogenesis of MetS [14]. The potential role of oxidative stress and chronic inflammation in MetS has also been reported, and increased oxidative stress or the presence of chronic inflammation may affect the development of MetS [15]–[17]. Despite the critical roles of insulin resistance and/or oxidative stress and chronic inflammation in MetS pathogenesis [14]–[17] and the functions of zinc related to insulin resistance [3], [4], oxidative stress [5], or chronic inflammation [6], [7], studies on the association between MetS and body zinc status are scarce and the results are controversial. In a cross-sectional study of Iranian participants, serum zinc levels were significantly higher in men with MetS as compared to those without MetS, but had a trend of a negative association in women with MetS [18]. In a study conducted in a Chinese population, Chinese men with MetS had a higher level of serum zinc [19]; however, serum zinc levels were not associated with MetS in European [20] or Persian populations [21]. Furthermore, no studies have been conducted on the association between MetS and serum zinc levels in a Korean population. Therefore, we evaluated whether serum zinc levels are associated with MetS and whether serum zinc levels differ according to MetS components in Korean adults using the data from KNHANES V-1.

## Materials and Methods

### Study population

We used data collected from the KNHANES V-1 conducted from January to December 2010. The KNHANES is implemented by the Korea Centre for Disease Control and Prevention (KCDC) during 3-year intervals to assess the status of public health and to provide baseline data for the development, establishment, and evaluation of public health policies in the Korean population. In KNHANES, participants comprise non-institutionalized individuals ≥1 year of age, selected using a stratified, multi-stage cluster probability sampling design to ensure an independent and homogeneous sampling each year in addition to a nationally representative sampling. Data are collected by a variety of means, including household interviews, anthropometric and biochemical measurements, and nutritional status assessments [22]. All the protocols were approved by the Institutional Review Board of the KCDC and the participants provided written informed consent at baseline.

In the KNHANES V-1, 10,938 participants were recruited, and 8,958 of them completed the survey (participation rate: 81.9%). In this cross-sectional study, we originally examined 1,988 adults ≥20 years of age by assessing serum zinc levels from data on 8,958 participants collected from KNHANES V-1. We excluded those participants with missing information or values for the major variables (n = 60) and with decreased kidney function (estimated glomerular filtration rate <30****mL/min/1.73 m^2^) (n = 2). The population for the current study thus consisted of 1,926 participants. The current study was approved by the Institutional Review Board of the Catholic University of Korea (IRB approval number: VC14EISI0070).

### Definitions of variables

We used the revised criteria of the National Cholesterol Education Program Adult Treatment Panel III (NCEP-ATP III) to define MetS [23]. The NCEP-ATP III criteria define MetS as the presence of any of three or more of the following five MetS components: waist circumference ≥90****cm (≥85****cm for women) according to the Korean Society for the Study of Obesity cut-off point for abdominal obesity [24]; triglyceride levels ≥150****mg/dL or taking medication for elevated triglycerides; high-density lipoprotein (HDL) cholesterol levels <40****mg/dL (<50****mg/dL for women) or taking medication to reduce HDL-cholesterol; systolic blood pressure ≥130****mmHg or diastolic blood pressure ≥85****mmHg or taking antihypertensive medication; fasting glucose levels ≥100****mg/dL or taking medication for elevated glucose levels. The MetS phenotypes represented any three or more combinations of the five MetS components. Serum zinc levels were categorized by quartiles with quartile 1 (Q1) representing the lowest zinc levels, Q2: low-medium zinc levels, Q3: high-medium zinc levels and Q4: the highest zinc levels.

### Laboratory measurements

Blood samples were collected from the antecubital vein of each participant after at least 12 h of fasting, processed, refrigerated immediately and transported in cold storage to the Central Testing Institute in Seoul, Korea. All blood samples were analyzed within 24 h after arrival at the testing facility. Fasting plasma glucose, triglyceride, HDL-cholesterol, and creatinine levels were measured using an auto-analyzer (Hitachi Automatic Analyzer 7600, Hitachi, Japan). Analysis of serum insulin was performed using an immunoradiometric assay (1470 WIZARD Gamma Counter, PerkinElmer, Finland). Insulin resistance was assessed using the homeostasis model assessment of insulin resistance (HOMA-IR) index, which was calculated as follows: [fasting glucose (mg/dL) × fasting insulin (µIU/mL)]/405 [25]. The glomerular filtration rate (GFR) was estimated by the re-expressed “Modification of Diet in Renal Disease” study equation using calibrated serum creatinine values [26]; the formula used for estimated GFR (eGFR) was as follows:
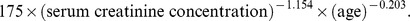



For measuring serum zinc concentrations, a trace element tube was used and serum zinc concentration was determined by inductively coupled plasma mass spectrometry using PerkinElmer ICP-MS (PerkinElmer, MA, USA). Serum samples were diluted with 2% nitric acid, and serum zinc concentration was obtained from a linear relationship (r = 0.999) between concentrations of zinc stock standard (1000 µg/mL, SPEX CertiPrep, NJ, USA) and absorbance. The accuracy of the analytical methods was tested with standard reference material (ClinChek Serum Controls, lyophilised for trace elements, RECIPE, Munich, Germany). The standard deviation index was 0.50, and coefficients of variation for inter- and intra-assay were 2%, and 4%, respectively.

### Clinical and anthropometric measurements

Anthropometric measurements of the participants were performed by specially trained examiners. Height and weight were measured after an overnight fast while the participants wore a lightweight gown, and waist circumference was measured using a measuring tape in the horizontal plane around the umbilical region after exhaling. Blood pressure measurements were taken in the sitting position after a rest period of at least 5 min. Body mass index (BMI) was calculated as each participant’s weight (in kilograms) divided by the square of height (in meters).

Self-reported information regarding age, gender, smoking, alcohol consumption, and the amount of physical activity were obtained. Cigarette smoking was divided into three categories based on current use estimates: non-smoker, ex-smoker and current smoker. Alcohol consumption was classified into three categories: abstinence (no alcoholic drinks consumed within the last year), moderate drinking (less than 14 standard drinks consumed for men or 7 for women per week) and heavy drinking (more than 14 standard drinks consumed for men or 7 for women per week). Physical activity was classified as low or not. Low physical activity was defined as 150 min or less of moderate intensity or 75 min or less of vigorous intensity exercise per week [27].

### Statistical analysis

To analyze the data using a complex sampling design, we used the SAS PROC SURVEY module, considering strata, clusters, and weights. All analyses were performed using the sample weights from KNHANES. Gender-specific characteristics of the study population were analyzed using independent t-tests for continuous variables and the chi-squared test for dichotomous variables. The data are expressed as means **±** standard errors or percentages and as geometric means and 95% confidence intervals (CI) for skewed distributions. Variables with skewed distributions were analyzed after logarithmic transformation. The correlations between serum zinc levels, MetS components and insulin resistance were analyzed using Pearson’s correlation analysis. The differences in the mean values of MetS components according to serum zinc level quartile were evaluated using analysis of covariance (ANCOVA) with age, smoking, alcohol consumption, physical activity, BMI, and eGFR levels as covariates. We also examined the relationship between serum zinc levels as the dependent variable and MetS components as the independent variable, using multiple logistic regression analysis. Model 1 was adjusted for age, and model 2 was adjusted for age, smoking, alcohol consumption, physical activity, BMI, and eGFR levels. Serum zinc levels and the percentage of participants in Q4 were analyzed according to the number of MetS components using ANCOVA after adjusting for the abovementioned covariates and using the chi-squared test, respectively. The percentages of participants according to MetS phenotypes and serum zinc levels were analyzed using the chi-square test. All statistical analyses were performed using the SAS software (ver. 9.2; SAS Institute, Cary, NC, USA). P-values<0.05 were considered to indicate statistical significance.

## Results

### 1. Characteristics of the participants according to serum zinc levels and the correlations between serum zinc levels and metabolic syndrome components

The present study was conducted using a total of 1,926 participants. In this population, the prevalence of MetS was 26.4% (n = 248) in men and 26.4% (n = 260) in women. Mean serum zinc levels in men with and without MetS were 142.0±2.4 µg/dL and 141.1±1.9 µg/dL (p = 0.717), respectively, and in women with and without MetS were 127.5±2.5 µg/dL and 129.6±1.9 µg/dL (p = 0.419), respectively. Table 1 shows the characteristics of the study participants according to serum zinc level quartiles, in particular, Q1-3 versus Q4. In men, significant differences in age and fasting glucose and insulin levels were observed according to serum zinc levels, while age, systolic blood pressure, and insulin levels were higher in women in serum zinc level Q1-3 than in Q4.

**Table 1:** Characteristics of the study participants according to serum zinc level quartile.

	Men			Women		
	Q1,2,3 (n = 704)	Q4 (n = 235)	p value	Q1,2,3 (n = 740)	Q4 (n = 247)	p value
Age (years)	44.8±0.9	41.2±1.0	0.009	46.4±0.8	42.7±1.3	0.012
Current smoking (%)	43.5	42.2	0.786	5.3	5.4	0.951
Heavy drinking (%)	9.1	9.7	0.811	6.3	6.5	0.932
Low physical activity (%)	72.0	68.7	0.425	81.2	78.7	0.526
Body mass index (kg/m^2^)	24.1±0.1	24.0±0.3	0.837	23.5±0.2	23.2±0.3	0.457
Zinc (µg/dL)	128.8±1.3	180.0±2.0	<0.001	118.2±1.2	166.9±2.0	<0.001
Metabolic syndrome (%)	27.1	24.5	0.509	27.1	24.1	0.479
Waist circumference (cm)	84.0±0.5	84.3±0.8	0.715	78.3±0.5	77.2±0.9	0.252
SBP (mmHg)	123.4±0.7	121.3±1.0	0.105	118.7±0.8	114.2±1.0	0.002
DBP (mmHg)	80.7±0.6	80.9±0.8	0.850	75.1±0.5	73.4±0.7	0.071
Fasting glucose (mg/dL)	100.5±1.5	95.1±1.3	0.004	95.1±1.0	92.4±1.1	0.081
Triglycerides (mg/dL)	127.0±1.0	127.5±1.1	0.654	95.7±1.0	93.6±1.1	0.333
HDL-cholesterol (mg/dL)	50.0±0.6	49.0±0.9	0.378	56.1±0.6	55.5±1.0	0.618
Insulin (µIU/mL)*	9.7 (9.4–10.1)	9.4 (8.8–10.0)	0.037	10.0 (9.7–10.4)	9.6 (9.0–10.2)	0.018
HOMA-IR index*	2.4 (2.3–2.4)	2.2 (2.0–2.4)	0.078	2.3 (2.2–2.4)	2.2 (2.0–2.3)	0.077
eGFR (mL/min/1.73 m^2^)	94.9±0.9	97.0±1.0	0.170	100.4±1.06	100.5±1.8	0.944

Values are expressed as means ± standard errors or percentages and as geometric means and 95% confidence intervals for skewed distributions*. Quartile 1 (Q1): the lowest zinc levels, Q2: low-medium zinc levels, Q3: high-medium zinc levels, and Q4: the highest zinc levels. SBP = systolic blood pressure; DBP = diastolic blood pressure; HDL = high-density lipoprotein; HOMA-IR = homeostasis model assessment of insulin resistance; eGFR = estimated glomerular filtration rate.

In both men and women, significant negative correlations were observed between serum zinc levels and fasting glucose (for men: r = −0.127, p = 0.003; for women: r = −0.078, p = 0.045) and the HOMA-IR index (r = −0.120, p = 0.003 for men, r = −0.113, p = 0.006 for women), and, in women, between serum zinc levels and systolic blood pressure (r = −0.082, p = 0.015) and insulin levels (r = −0.097, p = 0.023) (Table 2).

**Table 2:** Correlations between serum zinc levels and metabolic syndrome components.

	Correlation coefficient (r) with zinc level	
	Men	Women
Waist circumference	0.052	−0.070
Systolic blood pressure	−0.047	−0.082*
Diastolic blood pressure	0.013	−0.066
Fasting glucose	−0.127**	−0.078*
Triglycerides	0.020	−0.016
HDL-cholesterol	−0.033	−0.041
Insulin†	−0.066	−0.097*
HOMA-IR index†	−0.120**	−0.113**

*p<0.05,

**p<0.01.

† Variables with skewed distributions performed log-transformation.

HDL = high-density lipoprotein; HOMA-IR = homeostasis model assessment of insulin resistance.

### 2. Mean metabolic syndrome component values according to serum zinc level quartile

The mean values of MetS components adjusted for age, smoking, alcohol consumption, physical activity, BMI, and eGFR levels according to serum zinc level quartile are shown in Table 3. In men, as serum zinc levels increased, fasting glucose levels decreased (p for trend = 0.013). HDL-cholesterol levels were not significantly different according to quartiles of serum zinc levels in both men and women (p = 0.398 and 0.308, respectively), but as serum zinc levels increased, HDL-cholesterol levels showed a decreasing trend (p for trend = 0.088 and 0.083, respectively).

**Table 3:** Adjusted mean values of metabolic syndrome components according to serum zinc level quartile.

		Serum zinc levels					
		Q1	Q2	Q3	Q4	p value	p for trend
Men							
	N	234	235	235	235		
	Zinc (µg/dL)	107.6±11.0	130.5±4.5	147.7±5.5	180.3±23.8		
	Range of zinc level (µg/dL)	69.7–122.5	122.6–138.3	138.4–157.6	157.8–333.2		
	Waist circumference (cm)	83.3±0.7	84.4±0.8	84.5±0.8	84.9±0.8	0.487	0.168
	SBP (mmHg)	124.0±1.3	122.3±1.2	124.4±1.3	122.6±1.1	0.586	0.705
	DBP (mmHg)	80.9±1.1	80.6±0.9	80.7±0.9	80.9±0.8	0.993	0.997
	Fasting glucose (mg/dL)	104.7±2.9	99.2±1.3	98.0±2.8	96.8±1.3	0.063	0.013
	Triglycerides (mg/dL)	119.7±1.0	138.1±1.0	126.0±1.0	130.9±1.1	0.142	0.423
	HDL-cholesterol (mg/dL)	50.5±0.9	50.1±1.1	49.2±1.0	48.4±0.9	0.398	0.088
Women							
	N	246	248	246	247		
	Zinc (µg/dL)	99.6±9.3	119.7±4.3	137.3±5.4	167.9±24.0		
	Range of zinc level (µg/dL)	58.1–111.6	111.7–127.2	128.0–146.9	147.0–347.7		
	Waist circumference (cm)	77.8±0.8	78.2±0.6	77.1±0.8	77.6±0.8	0.764	0.644
	SBP (mmHg)	116.4±1.0	119.2±1.2	116.8±1.2	115.9±1.1	0.201	0.481
	DBP (mmHg)	74.4±0.7	75.0±0.7	74.8±0.9	73.9±0.8	0.793	0.672
	Fasting glucose (mg/dL)	94.2±1.0	97.2±2.5	91.5±1.0	93.2±1.2	0.074	0.080
	Triglycerides (mg/dL)	89.2±1.0	93.3±1.0	94.8±1.0	96.9±1.0	0.565	0.162
	HDL-cholesterol (mg/dL)	57.6±1.1	55.6±0.8	55.8±0.8	55.0±0.8	0.308	0.083

Values are means ± standard errors. Adjustment for age, smoking, alcohol consumption, physical activity, BMI, and eGFR levels. Quartile 1 (Q1): the lowest zinc levels, Q2: low-medium zinc levels, Q3: high-medium zinc levels, and Q4: the highest zinc levels. SBP = systolic blood pressure; DBP = diastolic blood pressure; HDL = high-density lipoprotein.

### 3. Associations between serum zinc levels and metabolic syndrome and its components

Unadjusted odds ratios (ORs), age-adjusted ORs (model 1), and multivariate-adjusted ORs (model 2) of serum zinc levels according to the presence of MetS and its components are shown in Table 4. Men with elevated fasting glucose levels were more likely to have low serum zinc levels than were those with normal fasting glucose levels (unadjusted OR 0.50, 95% confidence interval [CI] 0.33–0.77, p = 0.001), and this negative association remained significant after adjusting for covariates (adjusted OR 0.58, 95% CI 0.36–0.93, p = 0.023). The multivariate-adjusted OR of serum zinc levels for elevated triglyceride levels in men was 1.47 (95% CI 1.01–2.13, p = 0.044). However, no significant association between MetS components and serum zinc levels was found in women. No association was detected between the presence of MetS and serum zinc levels in either men or women.

**Table 4:** Odds ratios and 95% confidence intervals of serum zinc level according to metabolic syndrome and its components.

	Men			Women		
	OR	95% CI	p value	OR	95% CI	p value
Elevated waist circumference						
Unadjusted	1.17	0.76–1.81	0.472	0.92	0.63–1.34	0.660
Model 1	1.27	0.81–1.97	0.294	1.13	0.74–1.73	0.572
Model 2	1.28	0.82–1.98	0.279	1.15	0.76–1.74	0.875
Elevated blood pressure						
Unadjusted	0.98	0.70–1.37	0.890	0.62	0.42–0.92	0.018
Model 1	1.17	0.79–1.74	0.442	0.78	0.41–1.49	0.446
Model 2	1.12	0.74–1.68	0.591	0.78	0.42–1.44	0.420
Elevated fasting glucose						
Unadjusted	0.50	0.33–0.77	0.001	0.60	0.36–1.02	0.059
Model 1	0.58	0.36–0.92	0.020	0.70	0.40–1.24	0.222
Model 2	0.58	0.36–0.93	0.023	0.75	0.43–1.32	0.318
Elevated triglycerides						
Unadjusted	1.34	0.94–1.91	0.110	0.74	0.45–1.21	0.224
Model 1	1.48	1.02–2.14	0.040	0.87	0.54–1.40	0.574
Model 2	1.47	1.01–2.13	0.044	0.91	0.58–1.42	0.667
Reduced HDL-cholesterol						
Unadjusted	1.29	0.87–1.91	0.201	0.92	0.61–1.40	0.702
Model 1	1.40	0.93–2.09	0.106	1.09	0.72–1.63	0.693
Model 2	1.42	0.94–2.14	0.097	1.13	0.74–1.71	0.577
Metabolic syndrome						
Unadjusted	0.87	0.58–1.31	0.509	0.85	0.54–1.34	0.483
Model 1	1.02	0.66–1.59	0.921	1.13	0.67–1.91	0.643
Model 2	1.01	0.65–1.58	0.956	1.21	0.72–2.05	0.478

Model 1: adjustment for age, Model 2: adjustment for age, smoking, alcohol consumption, physical activity, BMI and eGFR levels. HDL = high-density lipoprotein; OR = odds ratio; CI = confidence interval.

### 4. Serum zinc levels and the percentage of the highest zinc level group (Q4) according to the number of metabolic syndrome components

**Figure 1:**
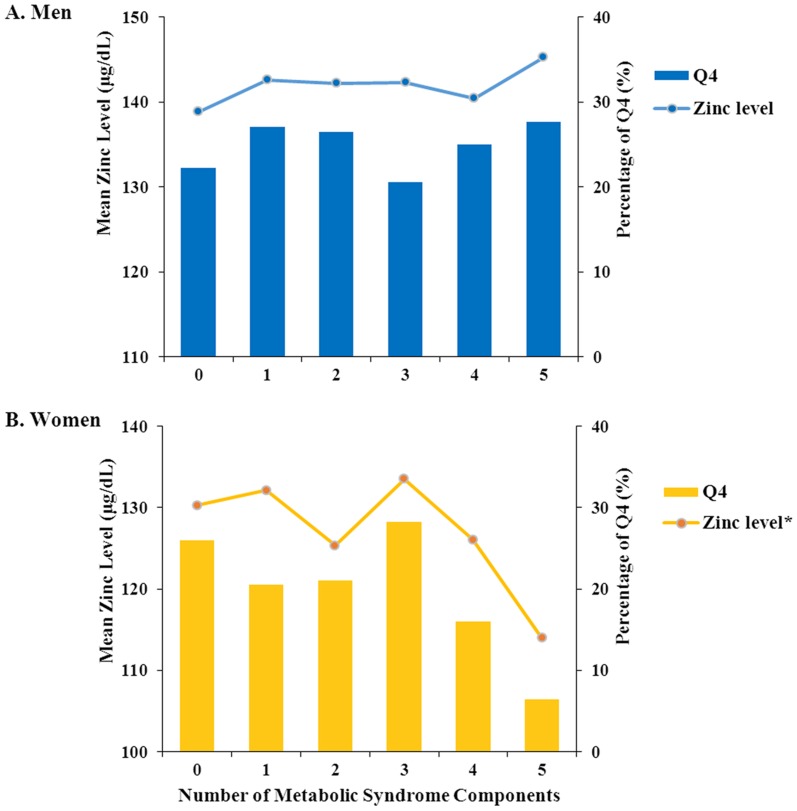
Serum zinc levels and the percentage of participants in the highest zinc level quartile according to the number of metabolic syndrome components. *p value<0.05.

Figure 1 shows mean serum zinc levels, and the percentage of the highest zinc level group (Q4) according to the number of MetS components. After adjusting for age, smoking, alcohol drinking, physical activity, BMI, and eGFR levels, in women, a difference in serum zinc levels was observed based on the number of MetS components (p = 0.002). Furthermore, in women with MetS (the number of MetS components: 3, 4 and 5), serum zinc levels showed a decreasing trend as the number of MetS components increased. In terms of the percentage of Q4, the difference showed according to number of MetS components, and the percentage of Q4 in women with MetS showed a decreasing trend as the number of MetS components increased (p = 0.050). No differences in mean serum zinc levels and the percentages of Q4 according to number of MetS components were observed in men (p = 0.727 and p = 0.741, respectively).

### 5. Percentages of participants according to the MetS component combinations (MetS phenotypes) and serum zinc levels

**Figure 2:**
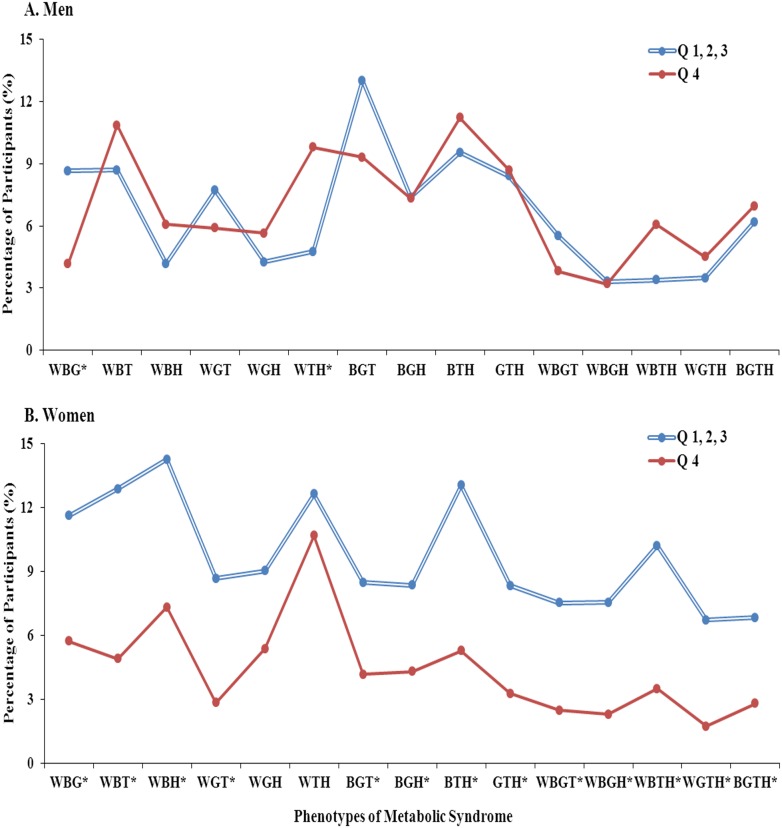
Participant distribution among metabolic syndrome phenotypes and serum zinc level quartiles. *p value<0.05, W: elevated waist circumference, B: elevated blood pressure, G: elevated fasting glucose, H: reduced HDL-cholesterol, T: elevated triglycerides.

The participant distribution according to the MetS component combinations and serum zinc levels (Q1-3, and Q4) is shown in Figure 2. In men with the MetS phenotype manifesting as increases in waist circumference, blood pressure and fasting glucose, the percentage of participants in Q4 was lower than in Q1-3 (p = 0.021); on the other hand, in men with the MetS phenotype manifesting as increased waist circumference, elevated triglyceride, and reduced HDL-cholesterol, the percentage in Q4 was higher than in Q1-3 (p = 0.012). There were no significant differences in the percentage of men with other MetS phenotypes. Among women of almost every MetS phenotype, the percentage of participants in Q4 was significantly lower than those in Q1-3, with the exception of the MetS phenotypes manifesting as increased waist circumference, elevated fasting glucose and reduced HDL-cholesterol, and as increased waist circumference, elevated triglycerides and reduced HDL-cholesterol.

## Discussion

We investigated the associations of serum zinc levels with MetS or its metabolic risk factors in Korean adults. The results of this study showed associations between serum zinc levels and certain MetS components. Serum zinc levels in men were negatively associated with elevated fasting glucose and positively associated with elevated triglycerides. In both men and women, as serum zinc levels increased, HDL-cholesterol levels showed a decreasing trend. Although there was no significant association found between serum zinc levels and the prevalence of MetS in either men or women, there were differences in serum zinc levels according to the number of MetS components in women; in particular, in women with MetS, serum zinc levels decreased as the number of MetS components increased, and low serum zinc levels (Q1-3) showed a greater prevalence than the highest serum zinc levels (Q4) among almost every MetS phenotype.

We used serum zinc concentration to assess body zinc status. Zinc status has been measured in a number of tissues such as serum or plasma, different blood cell types, hair, and nails [28]. However serum zinc concentration is viewed as the most appropriate indicator for evaluating individual’s zinc status, as compared to other assessment [29], although the sensitivity and specificity of the serum zinc level might be limited by responsiveness to confounding factors such as acute stress, infection, or altered steroidal hormone levels [30]. Furthermore serum zinc concentration is the only biomarker to show a dose-response relationship to dietary zinc manipulations [29]–[31], so mean serum zinc level in a population may reflect the status of dietary zinc intakes or zinc supplementation, and could be used as an indicator of zinc deficiency at the population level [30].

Several studies have reported a relationship between serum zinc levels and MetS. However, the relationship between serum zinc levels and the prevalence of MetS is inconclusive. In this study, no differences in serum zinc levels were seen between participants with and without MetS. In line with our findings, serum zinc levels did not differ significantly between subjects with and without MetS in a cohort study of 2,233 Iranian subjects aged 15–65 years [21], and in a cross-sectional study conducted in 1,902 European participants, serum zinc levels did not show an association with MetS [20]. However, Yu et al. [19] reported significantly higher serum zinc levels in subjects with MetS compared with those without MetS in a study of 379 Chinese men aged 24–57 years. Furthermore, in a population-based study consisting of 2,401 Iranian adults, mean serum zinc levels were positively associated with men with MetS compared with those without MetS; however, in women, medium serum zinc levels were associated with a lower prevalence of MetS, compared with the lowest zinc levels [18].

Because of the diversity of MetS phenotypes, the treatment strategies used for this condition may be different [32]. In men, serum zinc levels were negatively associated with elevated fasting glucose, but positively correlated with elevated triglycerides. Thus, the different directions of associations with serum zinc levels and certain components of MetS might account for the variations in serum zinc status according to the MetS phenotype (Figure 2A). In women, on the other hand, no significant association between MetS components and serum zinc levels was found, but with almost every MetS phenotype, the percentage of participants with the highest zinc level (Q4) was significantly lower than the percentage of those with the lowest or medium zinc level (Q1-3) (Figure 2B). Furthermore, in women with MetS, serum zinc levels showed a decreasing trend as the number of MetS components increased. Therefore, with regard to serum zinc levels in women, the presence or severity of MetS might be more useful than the MetS phenotype. Further investigations are warranted to clarify the gender difference in the association between serum zinc levels and MetS.

In this study, low serum zinc levels were associated with elevated fasting glucose levels in men, and significant negative correlations were found between serum zinc levels and fasting glucose as well as insulin resistance in both men and women. Similar to our findings, Islam et al. [33] reported that participants with pre-diabetes had lower zinc levels than did normal participants in a cross-sectional study of 280 Bangladesh adults aged ≥30 years, and Vashum et al. [34] showed that a higher serum zinc concentration was associated with increased insulin sensitivity in a cross-sectional study of 452 Australian adults aged 55–85 years. Insulin resistance is known to play a key role in the development of MetS, although the pathogenesis that unites the components of MetS is unclear. An overabundance of circulating fatty acids released by visceral fat might be a main contributor to the development of insulin resistance [35]. In an experimental study in rats, defects in insulin-stimulated tyrosine phosphorylation of insulin receptor substrates-1 and −2 by high levels of circulating fatty acids contributed to insulin resistance [36]. Zinc, however, is known to increase insulin receptor phosphorylation and downstream protein phosphorylation in insulin signaling pathways [1], [2], such that a decrease in body zinc status might cause insulin resistance [33], [34].

The issue of whether serum zinc levels are associated with plasma lipids is controversial. In agreement with our results, Ghasemi et al. [18] found a positive correlation between serum zinc levels and triglycerides in Iranian men whereas no association was observed between serum zinc concentrations and lipid profiles in a Kuwaiti population [37] or in Lebanese adults [38]. Although several studies have shown no association between serum zinc levels and HDL-cholesterol concentrations [18], [37], [38], we found a trend for a negative association between serum zinc and HDL-cholesterol levels in both men and women. Additionally, in a meta-analysis of 33 randomized controlled trials, no significant effects of zinc supplementation on serum lipids were observed, but zinc supplementation was associated with a significant decrease in HDL-cholesterol levels in a sub-group analysis of healthy participants, and HDL-cholesterol levels increased as a result of zinc supplementation in a sub-group analysis of subjects with type 2 diabetes mellitus [39]. However, the negative association between serum zinc and lipoprotein metabolism in our study should be considered cautiously, including the influence of zinc-rich foods such as red meat on plasma lipids [40] and various health conditions known to influence zinc homeostasis [41]–[44], and further investigations considering these factors are warranted to confirm the association between serum zinc levels and lipid profiles.

Other factors not included in the clinical definition of MetS, such as chronic inflammation [16] or oxidative stress [15], may lead to the development of MetS. Inflammatory cytokines released by visceral fat [45], including tumor necrosis factor-α (TNF-α), interleukin-6 (IL-6) and plasminogen activator inhibitor-1 (PAI-1), stimulate C-reactive protein (CRP) production in the liver, and these processes are associated with MetS [17]. On the other hand, oxidative stress, which occurs when reactive oxygen species (ROS) exceed the antioxidant capacity, may play an important role in MetS [15]. Zinc reduces inflammatory cytokine production via up-regulation of a zinc-finger protein, which inhibits nuclear factor-κB (NF-κB) activation [6], [7]. Furthermore, zinc, a cofactor for antioxidant enzymes, such as superoxide dismutase and glutathione peroxidase, decreases ROS generation and induces metallothionein, which decreases the **^.^**OH burden [2], suggesting that a decrease in body zinc status may contribute to the development or aggravation of MetS. In addition, chronic inflammation or oxidative stress may contribute to the decreased serum zinc levels. In experimental study with mice, the expression of zinc transporter gene, which plays a critical role in the hypozincemia, was up-regulated by inflammation [46], and in an experiment with HL-60 cells, the oxidative stress released zinc from metallothionein, one of cellular zinc buffering systems, and increased the availability of cellular zinc, therefore, it resulted in a cellular zinc deficiency [47], suggesting that as part of MetS, chronic inflammation or oxidative stress negatively affect to body zinc status, and then, the decreased zinc status may play an important role in the development or aggravation of MetS. To date, studies on the effects of zinc intake in adults with MetS are scarce, and have reported inconsistent results; therefore, further studies on the dietary intake or supplementation of zinc are warranted to associate with the status of chronic inflammation or oxidative stress, and to help reduce the prevalence or improve the manifestation of MetS.

The strengths of this study were that the data were collected through a representative nationwide survey of the South Korean population and that this is the first study in Korean adults to investigate the associations among serum zinc levels, MetS, and its metabolic risk factors. However, this study had certain limitations. First, it was conducted using a cross-sectional design. Second, dietary patterns and the kind of food as sources of zinc intake were not included as a covariate in this study, because dietary zinc intake was not estimated in the KNHANES. Zinc is highly concentrated in the organs and flesh of mammals, fowl, fish, and crustaceans. However, the bioavailability of zinc is determined mostly by the amount of zinc in the diet and phytate, which is a major inhibitor of zinc absorption, so not only total zinc intake but also the types of foods or dietary patterns should be considered when assessing dietary zinc intake [48]. Although zinc intake in the South Korean population was adequate in the data provided by 188 countries to the Food and Agriculture Organization of the United Nations [49], further studies are warranted to clarify the association between dietary zinc intake, serum zinc levels that represent body zinc status, and MetS. Third, we did not consider other trace elements, such as copper, that might affect serum zinc levels, since the trace elemental minerals were not measured in the KNHANES.

In conclusion, serum zinc levels were negatively associated with elevated fasting glucose levels and positively associated with elevated triglycerides in men. Serum zinc levels differed according to the number of MetS components in women, and serum zinc levels showed a decreasing trend as the number of MetS components increased in women with MetS. These findings suggest that serum zinc levels might be associated with MetS or metabolic risk factors. Further gender-specific studies are needed to evaluate the effects of dietary or supplemental zinc intake on the improvement of MetS.
